# How symptoms of prolonged grief disorder, posttraumatic stress disorder, and depression relate to each other for grieving ICU families during the first two years of bereavement

**DOI:** 10.1186/s13054-022-04216-5

**Published:** 2022-11-01

**Authors:** Fur-Hsing Wen, Holly G. Prigerson, Wen-Chi Chou, Chung-Chi Huang, Tsung-Hui Hu, Ming Chu Chiang, Li-Pang Chuang, Siew Tzuh Tang

**Affiliations:** 1 Department of International Business, Soochow University, Taipei, Taiwan, R.O.C.; 2 Department of Medicine, Weill Cornell Medicine, New York City, NY USA; 3 Division of Hematology-Oncology, Chang Gung Memorial Hospital at Linkou, Tao-Yuan, Taiwan, R.O.C.; 4 College of Medicine, Chang Gung University, Tao-Yuan, Taiwan, R.O.C.; 5 Department of Internal Medicine, Division of Pulmonary and Critical Care Medicine, Chang Gung Memorial Hospital at Linkou, Tao-Yuan, Taiwan, R.O.C.; 6 Department of Respiratory Therapy, Chang Gung University, Tao-Yuan, Taiwan, R.O.C.; 7 Department of Internal Medicine, Division of Hepato-Gastroenterology, Chang Gung Memorial Hospital at Kaohsiung, Kaohsiung, Taiwan, R.O.C.; 8 Department of Nursing, Chang Gung Memorial Hospital at Kaohsiung, Kaohsiung, Taiwan, R.O.C.; 9 School of Nursing, Medical College, Chang Gung University, 259 Wen-Hwa 1St Road, Kwei-Shan, Tao-Yuan, 333 Taiwan, R.O.C.; 10 Department of Nursing, Chang Gung University of Science and Technology, Tao-Yuan, Taiwan, R.O.C.

**Keywords:** PGD, PTSD, Depression, Comorbidity, Temporal relationships, ICU care, End-of-life care, Family members

## Abstract

**Background:**

Bereaved ICU family surrogates are at risk of comorbid prolonged grief disorder (PGD), posttraumatic stress disorder (PTSD), and depression. Knowledge about temporal relationships between PGD, PTSD, and depression is limited by a lack of relevant studies and diverse or inappropriate assessment time frames given the duration criterion for PGD. We aimed to determine the temporal reciprocal relationships between PGD, PTSD, and depressive symptoms among ICU decedents’ family surrogates during their first 2 bereavement years with an assessment time frame reflecting the PGD duration criterion.

**Methods:**

This prospective, longitudinal, observational study examined PGD, PTSD, and depressive symptoms among 303 family surrogates of ICU decedents from two academic hospitals using 11 items of the Prolonged Grief Disorder-13, the Impact of Event Scale—Revised, and the depression subscale of the Hospital Anxiety and Depression Scale, respectively, at 6, 13, 18, and 24 months post-loss. Cross-lagged panel modeling was conducted: autoregressive coefficients indicate variable stability, and cross-lagged coefficients indicate the strength of reciprocal relationships among variables between time points.

**Results:**

Symptoms (autoregressive coefficients) of PGD (0.570–0.673), PTSD (0.375–0.687), and depression (0.591–0.655) were stable over time. Cross-lagged standardized coefficients showed that depressive symptoms measured at 6 months post-loss predicted subsequent symptoms of PGD (0.146) and PTSD (0.208) at 13 months post-loss. PGD symptoms did not predict depressive symptoms. PTSD symptoms predicted subsequent depressive symptoms in the second bereavement year (0.175–0.278). PGD symptoms consistently predicted subsequent PTSD symptoms in the first 2 bereavement years (0.180–0.263), whereas PTSD symptoms predicted subsequent PGD symptoms in the second bereavement year only (0.190–0.214). PGD and PTSD symptoms are bidirectionally related in the second bereavement year.

**Conclusions:**

PGD, PTSD, and depressive symptoms can persist for 2 bereavement years. Higher PGD symptoms at 6 months post-loss contributed to the exacerbation of PTSD symptoms over time, whereas long-lasting PTSD symptoms were associated with prolonged depression and PGD symptoms beyond the first bereavement year. Identification and alleviation of depression and PGD symptoms as early as 6 months post-loss enables bereaved surrogates to grieve effectively and avoid the evolution of those symptoms into long-lasting PGD, PTSD, and depression.

**Supplementary Information:**

The online version contains supplementary material available at 10.1186/s13054-022-04216-5.

## Introduction

The impact on families of ICU bereavement, an increasingly [1] frequent [2] loss experience in the post-COVID-19 era [3], can be severe and prolonged. For a minority of persons, bereavement may precipitate an umbrella of severe, disturbed grief reactions, including symptoms of prolonged grief disorder (PGD) [4], posttraumatic stress disorder (PTSD) [5], and depression [6]. Distressed psychological grief reactions more often co-occur than not [7, 8]. If after ICU death at-risk family members go unnoticed, healthcare professionals miss opportunities to treat or prevent psychological distress, leading to unnecessary suffering.

Understanding the temporal relationships between grief-related psychological distress may help disentangle the mechanism of mental comorbidity [9]. Early grief-related symptoms may contribute to other grief reactions in later stages. Identifying psychological distress promptly in the preceding period and targeting it with appropriate symptom management may block subsequent development/exacerbation of long-lasting comorbid psychological distress and functional impairment. Yet, knowledge about temporal relationships between PGD, PTSD, and depression is limited with most of the focus on PTSD and depression [10–16], less on PGD and PTSD [17–20], and least on PGD and depression [19, 21, 22]. Only one study [19] was found to explore the temporal relationships between PGD, PTSD, and depression simultaneously following traumatic losses from an airplane crash. Indeed, temporal relationships between PGD, PTSD, and depression cannot be deduced from studies that only examined reciprocal relationships between two psychological grief-related responses.

Importantly, methodological inconsistencies were identified in these studies [10–22]. In previous studies [10–22], 2–4 waves of assessments were collected across periods from 0.5–7 months [11, 22] to 18–35 years post-event [13]. Such heterogeneity makes comparison challenging. Further, PGD is defined as a spectrum of grief symptoms occurring at least 6 months after the death as per the minimum duration criterion [23]. Assessments made earlier (1–4 [22] or 4–5 [20] months post-loss) may invalidate the PGD assessment. Therefore, the purpose of this study was to establish an appropriate time interval reflecting the PGD duration criterion and longitudinally determine the temporal reciprocal relationships between symptoms of PGD, PTSD, and depression among ICU family surrogates during their first 2 bereavement years to determine a potential mechanism of comorbidity.

## Materials and methods

### Study design/setting/study participants

Data for this study came from a longitudinal, observational study on associations between quality of end-of-life (EOL) ICU care and family surrogates’ bereavement outcomes, including symptoms of anxiety [24], depression [24], and PTSD [25]. Information on sampling strategy, patient and family characteristics, and study settings was reported [24, 25]. At two academically affiliated Taiwanese hospitals, a consecutive sample was recruited from family surrogates responsible for decision-making for critically ill patients at high risk of death (APACHE II scores > 20) in level III medical ICUs staffed by intensivists from January 2018 to March 2020 and followed through July 2022. Each surrogate signed informed consent for participation and review of the patient’s medical record. The study site’s research ethics committee approved this study (201700343B0).

### Data collection

Participant characteristics were collected through a review of medical records and surrogate interviews at enrollment. Subsequent phone interviews to assess surrogate psychological distress at 1, 3, 6, 13, 18, and 24 months post-loss complied with the minimum duration criterion for PTSD (≥ 1 month) [26] and avoided the anniversary effect at 12 months post-loss. Only data collected since 6 months post-loss were used in this study to accommodate the PGD duration criterion [23].

### Measures

*PGD symptoms* were measured by 11 items of the Prolonged Grief Disorder-13 [23]. The PG-11 includes one separation distress symptom, nine cognitive and emotional symptoms, and one functional impairment symptom but excludes 2 dichotomous items regarding duration and impairment criteria which measure dimensions other than grief symptoms. Participants rate how often symptoms occurred in the preceding month on a 5-point scale (1 = never, 5 = always).

*PTSD symptoms* were measured by the 22-item Impact of Event Scale—Revised (IES-R) [26], a screening instrument for PTSD symptoms on three subscales: intrusion, avoidance, and hyperarousal. For each item, participants rate their PTSD symptom distress level during the preceding week on a 0 (not at all)-4 (extremely) Likert scale [26].

*Depressive symptoms* were measured by the seven-item depression subscale of the Hospital Anxiety and Depression Scale (HADS-D) [27].HADS-D total scores range from 0 to 21 [27].

### Data analysis

A series of cross-lagged panel modeling [28] (CLPM) was conducted to examine the temporal reciprocal relationships between PGD, PTSD, and depressive symptoms across the four assessment waves (6–24 months post-loss) by structural equation modeling in Mplus 8.6. CLPM is a method for describing the reciprocal relationships among multiple variables between a series of timepoints. The autoregressive coefficient quantifies inter-individual stability for a specific variable (e.g., do PGD symptoms at Tn predict PGD symptoms at Tn + 1), whereas the cross-lagged coefficient quantifies the association between the prior score of one variable and the subsequent scores of the other variables (e.g., do PGD symptoms at Tn predict PTSD or depressive symptoms at Tn + 1), thereby indicating the temporal relationship between two variables [28, 29]. Cross-lagged coefficients are calculated while controlling for autoregressive coefficients and for correlations among the examined variables at each wave [28, 29].

To obtain the most parsimonious CLPM, the statistical fit of an unconstrained model (Model 1) was compared with constrained models [19]: Model 2 assumed the autoregressive paths for each symptom variable are equal, Model 3 assumed both the autoregressive and the cross-lagged paths are equal, and Model 4 included Model 3 assumptions and assumed associations among the three symptom variables are consistent across each wave. Model fit was assessed by the following indices: (1) comparative fit index (CFI) > 0.90 [30], (2) Tucker–Lewis index (TLI) > 0.90 [30], (3) root-mean-square error of approximation (RMSEA) < 0.10 [31], (4) standardized root-mean-square residual (SRMR) < 0.05 [32], and (5) smallest Akaike information criterion (AIC), Bayesian information criterion (BIC), and sample-size adjusted BIC (SABIC) [33]. We selected the best-fitting model and controlled suggested covariates [19]: gender (0: female, 1: male), kinship to deceased (0: others, 1: spouse, 2: adult child), and educational level (0: ≤ senior high school, 1: > senior high school). Missing data on symptom levels of PGD, PTSD, and depression were handled using full information maximum likelihood (FIML) estimation which produces the least biased estimates of missing values among other imputation methods [34]. We adopted benchmark values synthesized for cross-lagged effects of CLPM: 0.03 (small effect), 0.07 (medium effect), and 0.12 (large effect) [35]. All significance tests were two-tailed at *p* < 0.05 level.

## Results

### Participant characteristics

**Fig. 1:**
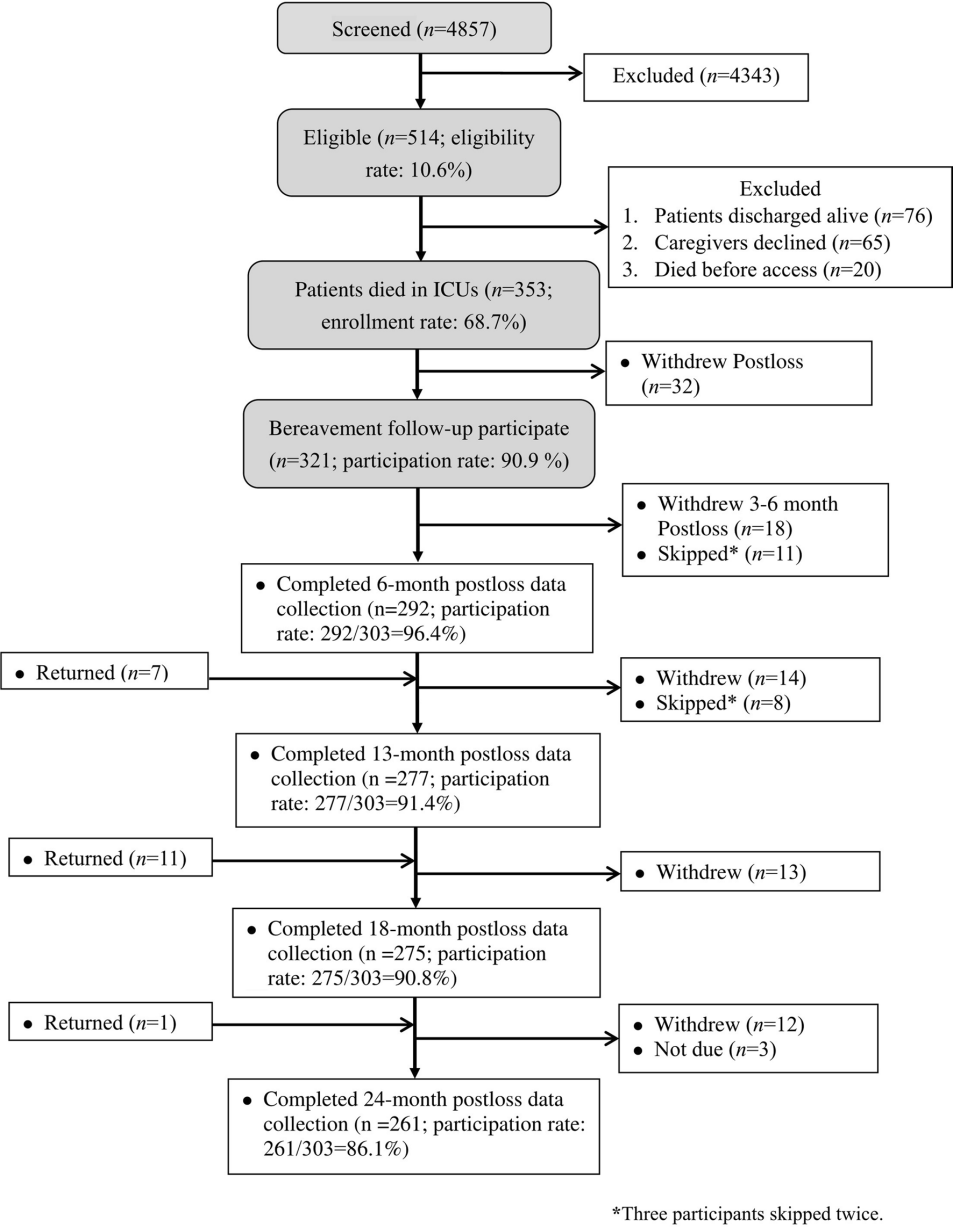
Participant flowchart

Among the 353 patients who died in the ICUs, 321 family surrogates (90.9%) participated in bereavement surveys (Fig. 1). Of them, 303 (94.4%) provided data 6–24 months post-loss and constituted the study sample; 292, 277, 275, and 261 surrogates completed surveys at 6, 13, 18, and 24 months post-loss, respectively (Fig. 1). The reasons for attrition are detailed in Fig. 1. Characteristics of the sample and their relatives are in Tables 1 and 2, respectively. Surrogates (Additional file 1: Table S1, Additional file 2: Table S2) who completed, withdrew, or skipped surveys since 6 months post-loss and their respective relative (Additional file 3: Table S3) did not significantly differ. Examination of missing symptom data by FIML [34] showed no examinations were significant (Little’s missing completely at random testing [*χ*^2^/degree of freedom, p value] for PGD [30.557/20, *p* = 0.061], PTSD [12241/19, *p* = 0.875], and depression [26.303/19, *p* = 0.122]), indicating data were missing completely at random. Thus, analyses in this study were based on the total sample.

**Table 1:** Comparison between surrogates who did or did not participate in 6–24 months bereavement surveys at enrollment (*N* = 353)

Variable	Participants (*n* = 303)	Declined bereavement surveys (*n* = 50)	*p*
*Age, n (%)*			.427
21–45	121 (39.9%)	23 (46.0%)	
46–55	86 (28.4%)	12 (24.0%)	
56–65	58 (19.1%)	12 (24.0%)	
> 65	38 (12.5%)	3 (6.0%)	
*Gender, n (%)*			.556
Male	126 (41.6%)	18 (36.0%)	
Female	177 (58.4%)	32 (64.0%)	
*Marital status, n (%)*			.135
Single	67 (22.1%)	12 (24.0%)	
Married/Cohabiting	228 (75.2%)	34 (68.0%)	
Separated/Widowed	8 (2.6%)	4 (8.0%)	
*Educational level, n (%)*			.238
> Senior high school	151 (49.8%)	30 (60.0%)	
≦ Senior high school	152 (50.2%)	20 (40.0%)	
*Financial status, n (%)*			.168
Making ends meet	255 (84.2%)	38 (76.0%)	
Financial strain	42 (13.9%)	9 (18.0%)	
Other	6 (2.0%)	3 (6.0%)	
*Relationship, n (%)*			.395
Spouse	88 (29.0%)	13 (26.0%)	
Child	166 (54.8%)	32 (64.0%)	
Other	49 (16.2%)	5 (10.0%)	
*Chronic disease, n (%)*			.160
Yes	107 (35.3%)	12 (24.0%)	
No	196 (64.7%)	38 (76.0%)	
*Living with the patient, n (%)*			.761
Yes	202 (66.7%)	31 (63.3%)	
No	101 (33.3%)	18 (36.7%)	

Variable	Participants (*n* = 303)	Withdrew from bereavement surveys (*n* = 50)	*p*
*Hospitalization for mental health problems, n (%)*			1.000
Yes	0 (0.0%)	0 (0.0%)	
No	302(100.0%)	50 (100.0%)	
*Hospitalization for medical problems, n (%)*			.246
Yes	14 (4.6%)	0 (0.0%)	
No	289 (95.4%)	50 (100.0%)	
*Emergency room visit, n (%)*			1.000
Yes	20 (6.6%)	3 (6.0%)	
No	283 (93.4%)	47 (94.0%)	
*Medication use for pain problems, n (%)*			.354
Yes	35 (11.6%)	3 (6.0%)	
No	268 (88.4%)	47 (94.0%)	
*Medication use for anxiety problems, n (%)*			.516
Yes	8 (2.6%)	0 (0.0%)	
No	295 (97.4%)	50 (100.0%)	
*Medication use for depressive problems or other psychiatric disturbances, n (%)*			1.000
Yes	3 (1.0%)	0 (0.0%)	
No	300 (99.0%)	50 (100.0%)

**Table 2:** Comparison between patients whose family surrogate did or did not participate in 6–24 months bereavement surveys (*N* = 353)

Variable, *n* (%)	Participated (*n* = 303)	Declined (*n* = 50)	*p*
*Gender*			.455
Male	192 (63.4%)	35 (70.0%)	
Female	111 (36.6%)	15 (30.0%)	
*Diagnosis*			.600
Cancer	149 (49.2%)	31 (62.0%)	
Chest	22 (7.3%)	2 (4.0%)	
Cardiovascular	14 (4.6%)	3 (6.0%)	
Digestive	11 (3.6%)	1 (2.0%)	
Kidney	16 (5.3%)	2 (4.0%)	
Other	91 (30.0%)	11 (22.0%)	
*Acute symptoms/problems at admission*			.344
Respiratory failure/distress	157 (51.8%)	26 (52.0%)	
Infection	85 (28.1%)	14 (28.0%)	
Shock	24 (7.9%)	1 (2.0%)	
Bleeding	10 (3.3%)	2 (4.0%)	
Cardiac arrest	11 (3.6%)	1 (2.0%)	
Others	16 (5.3%)	6 (12.0%)	
*Comorbidity*			.625
Yes	260 (85.8%)	41 (82.0%)	
No	43 (14.2%)	9 (18.0%)	

Variable, mean (SD)			
Age (years)	66.72 (14.37)	65.96 (10.86)	.666
APACHE^a^	28.30 (5.37)	28.68 (5.73)	.663
SOFA^a^	12.38 (4.04)	12.24 (4.07)	.823
Length of ICU stay (days)	21.06 (15.37)	21.58 (13.19)	.800
Time from ICU admission to enrollment (days)	14.78 (12.75)	14.52 (8.36)	.855
Time from enrollment to death (days)	7.28 (8.49)	8.06 (9.65)	.593

^a^Measured at enrollment

### Longitudinal temporal relationships between symptoms of PGD, PTSD, and depression

**Fig. 2:**
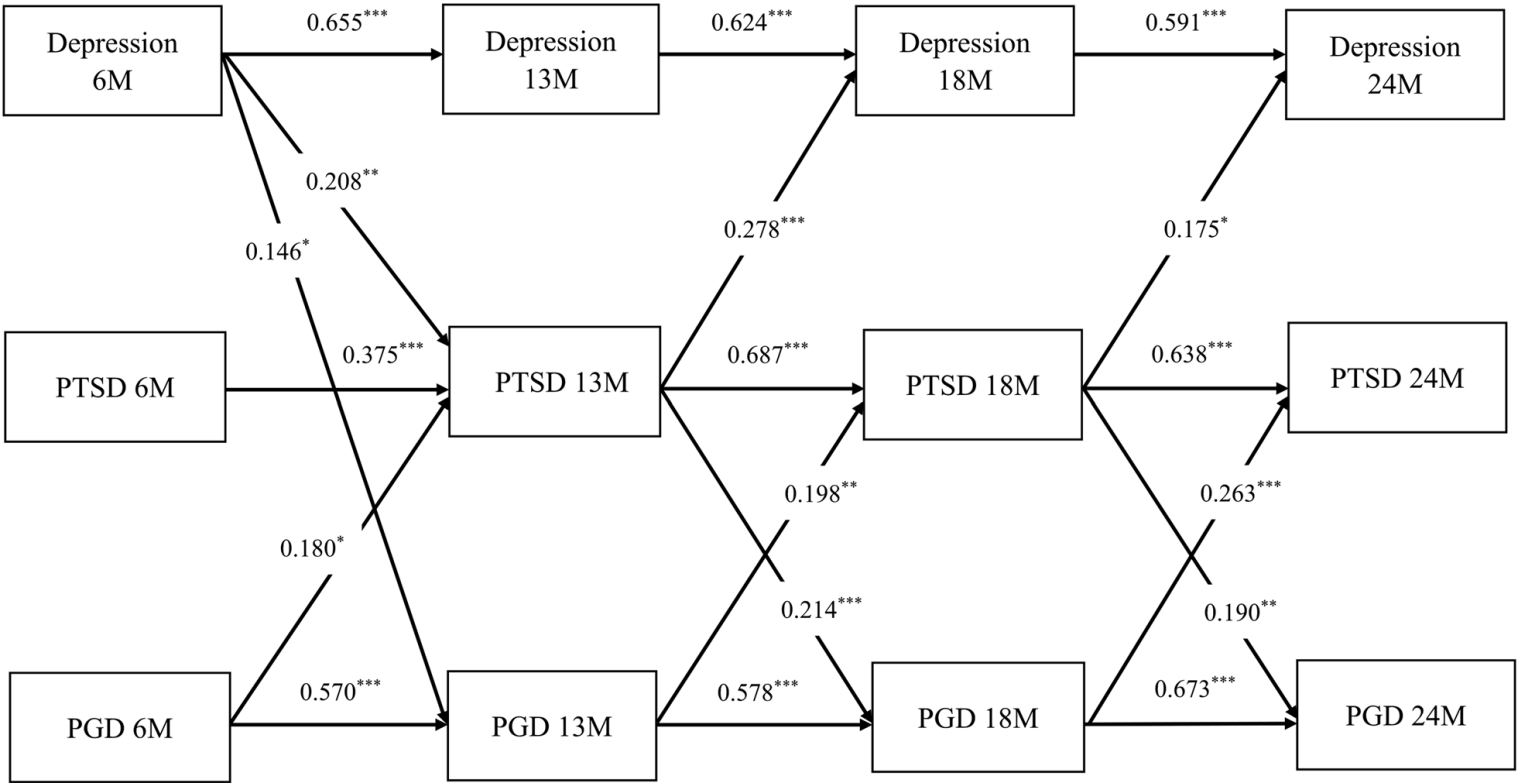
Significant reciprocal relationships between symptoms of depression, PTSD, and PGD by cross-lagged panel modeling from 6 to 24 months post-loss. Depressive symptoms were measured with the depression subscale of the Hospital Anxiety and Depression scale. PTSD symptoms were measured by the Impact of Event Scale—Revised. PGD symptoms were measured by 11 items of the Prolonged Grief Disorder-13. Autoregressive coefficients along horizontal arrows indicate stability of each variable, and cross-lagged coefficients along diagonal arrows indicate strength of temporal relationships between symptoms with autoregressive coefficient controlled. Cross-lagged effect sizes: 0.03 (small effect), 0.07 (medium effect), and 0.12 (large effect). Gender, kindship to the deceased, and education level were adjusted. *p < .05; **p < .01; ***p < .001

The unconstrained model was the best-fitting model (Table 3) with data fitting fairly well: CFI = 0.967, TLI = 0.921, and SRMR = 0.047, except for RMSEA = 0.131. Autoregressive standardized coefficients for PGD (0.570–0.673), PTSD (0.375–0.687), and depression (0.591–0.655) symptoms were remarkably stable (Fig. 2).

**Table 3:** Model fit indexes for different models of temporal reciprocal relationships between PGD, PTSD, and depressive symptoms

Model	Sample size	*Χ*^2^	# of parameter	AIC	BIC	SABIC	RMSEA	CFI	TLI	SRMR
Model 1	303	**167.687**	63	**15684.632**	15918.598	**15718.795**	0.131	**0.967**	0.921	**0.047**
Model 2	303	191.928	57	15696.873	15908.556	15727.782	0.126	0.963	0.927	0.049
Model 3	303	216.584	45	15697.529	**15864.647**	15721.931	**0.112**	0.960	**0.942**	0.079
Model 4	303	302.273	51	15771.318	15916.154	15792.466	0.128	0.942	0.925	0.072

Bold indicates the best fitting

Model 1: Unconstrained model

Model 2: Constrained the autoregressive paths to be equal

Model 3: Constrained the autoregressive and cross-lagged effects to be equal

Model 4: Constrained the autoregressive paths, the cross-lagged paths, and the associations between the three psychological distress symptom constructs measured at the same wave to be equal across the waves

*AIC* Akaike information criterion, *BIC* Bayesian information criterion, *SABIC* sample-size adjusted BIC, *RMSEA* root-mean-square error of approximation, *CFI* comparative fit index, *TLI* Tucker–Lewis index, *SRME* standardized root-mean-square residual

Cross-lagged standardized coefficients showed that depressive symptoms measured at 6 months post-loss predicted subsequent symptoms of PGD (0.146) and PTSD (0.208) at 13 months post-loss (Fig. 2). PGD symptoms did not predict depressive symptoms. PTSD symptoms predicted subsequent depressive symptoms in the second bereavement year (0.175–0.278). PGD symptoms consistently predicted subsequent PTSD symptoms in the first 2 bereavement years (0.180–0.263), whereas PTSD symptoms predicted subsequent PGD symptoms in the second bereavement year only (0.190–0.214). PGD and PTSD symptoms are bidirectionally related in the second bereavement year. All cross-lagged standardized coefficients (0.146–0.278) had a large effect.

## Discussion

Our study unprecedently investigated temporal relationships between PGD, PTSD, and depressive symptoms among ICU bereaved surrogates by CLPM. Most studies [10–22] on temporal relationships between PGD, PTSD, and/or depression examined survivors of natural disasters [12, 14–16] or anthropogenic traumatic events [10, 11, 13]. Studies on bereavement explored loss from natural disease [17, 21, 22] or pure/mixed unnatural disasters [18–20]. Temporal relationships between PGD, PTSD, and/or depression during grieving likely differ by the context of loss. This study contributes several novel observations on bereaved ICU surrogate grief reactions that implicate clinical practice.

### PTSD and depressive symptoms are temporally related

Depressive symptoms measured at 6 months post-loss predicted PTSD symptoms at 13 months post-loss (Fig. 2), whereas long-lasting PTSD symptoms predicted depressive symptoms beyond the first bereavement year. Literature on the temporal relationship between symptoms of PTSD and depression in the first year is inconclusive: depressive symptoms predicted subsequent PTSD symptoms between 1 and 3 months posttraumatic accidents [11]; and PTSD symptoms predicted subsequent depressive symptoms in 1.5–12 months after a tornado [15] or earthquake [16]. For the second year, previous results are consistent with ours: more pervasive PTSD symptoms predicted depressive symptoms at 24 months post-earthquake [16]. Results beyond 2 years post-event contrast with ours: depressive symptoms predicted PTSD symptoms 17–35 years post-combat [13], and PTSD and depression were bidirectionally related among bereaved about 1–4 years post-airplane crash [19]. Further research is needed to understand how the ICU context influences the temporal relationship between symptoms of depression and PTSD in the first 2 bereavement years.

A depressogenic model theorizes how depression leads to PTSD [11]. At the entry into bereavement, permanent separation from an important source of emotional support and interpersonal security [36] triggers separation distress—missing the deceased and grief over an impossible physical reunion [37, 38]. Compounded with doubt [39] and inadequate or conflicting social support during ICU EOL care decision-making [40, 41], surrogates may experience negative self-perceptions, helplessness, anhedonia, or negative worldview. Consequently, these depressive symptoms may hinder bereaved surrogates’ motivation and ability to manage exposure to trauma-related stimuli, leading to cognitive and behavioral avoidance [42] of trauma-related reminders, especially around the anniversary.

Stress sensitivity [43] may also predispose depressed, bereaved surrogates to PTSD symptoms, especially approaching the anniversary of loss. External stressors, like loss, can leave individuals with neurobiological vulnerabilities that increase sensitivity to subsequent stressors [43], like memories of the ICU-care experience evoked by the anniversary [28, 37]. Sensitization to this circumstance-related distress [28, 37] may elicit excessive cognitive or behavioral avoidance of loss reminders, thereby increasing bereaved surrogates’ vulnerability to PTSD symptoms at 13 months post-loss [38, 44].

Avoidance that stalls processing and acceptance of loss [38, 44] can entrench PTSD symptoms beyond the anniversary and subsequently predict surrogates’ depressive symptoms in the second bereavement year (Fig. 2). A demoralization model [11] and internalized trauma [37, 38, 44] connect PTSD to subsequent depression. Internalized trauma feels like excessive guilt and self-blame (as after EOL-care decisions like withdrawing or withholding life-sustaining treatments), demoralization, meaninglessness, and negative beliefs about oneself, the world, and the future [37, 38, 44]. Thus, long-lasting PTSD symptoms preceding prolonged depressive symptoms in the second bereavement year may indicate internalized trauma.

### Depressive symptoms predict PGD symptoms in the first year

Depressive symptoms measured at 6 months post-loss predicted PGD symptoms at 13 months post-loss (Fig. 2). Previously, no reciprocal relationships were observed between PDG and depression from approximately 1–4 years of bereavement [19] while other studies on loss from protracted advanced illness, often during hospice/palliative care, [21, 22] were inconsistent. Temporal relationships between PGD and depression were bidirectional at 1–4 months post-loss [22] while PGD predicted subsequent depression at 4–7 [22] and 6–24 [21] months post-loss. Future research should consider controlling characteristics of death (expected vs unexpected, chronic vs acute, clinical context) to determine their influence on different temporal relationships between PGD and depression.

We speculate that separation distress [37, 38] during early bereavement might sensitize depressed surrogates to loss reminders from the forthcoming anniversary [43], bringing on characteristic PGD symptoms: yearning for the eternally separated deceased, frequent preoccupying thoughts and memories of the deceased person, a feeling of disbelief or inability to accept the loss, and difficulty imagining a meaningful future without the deceased. The absence of social support may also prompt PGD symptoms, given the fragility of interpersonal relationships among those with profound depression [45]. With reluctance to accept outside support, isolation and loneliness may evolve into characteristic PGD symptoms like difficulty trusting people and feeling distant from others.

### PGD symptoms predict PTSD symptoms; PGD and PTSD symptoms are bidirectional in the second year

PGD symptoms consistently preceded PTSD symptoms over the first 2 bereavement years (Fig. 2), consistent with three previous studies [19, 20, 22]. Separation distress [37, 38] and stress sensitization theory [43], as described above, may similarly explain why PGD symptoms consistently predicted heightened PTSD symptoms. Furthermore, from the perspective of cognitive behavioral models of PGD [46], persistent yearning for the deceased and difficulties accepting the loss may interfere with processing and managing exposure to trauma-related reminders, leading to cognitive and behavioral avoidance and the development of PTSD symptoms. Difficulty trusting others, prolonged withdrawal from meaningful social activities, and meaninglessness in life may further worsen PTSD symptoms.

We observed that symptoms of PTSD predicted subsequent PGD symptoms beyond the first bereavement year, consistent with the report for survivors of a terrorist attack [20]. A meta-analysis indicated the prevalence of PGD is much higher (49% [95% confidence interval (CI) 33.6, 65.4]) in people bereaved by unnatural or traumatic deaths [47] than by losses primarily due to diseases (9.8% [95% CI 6.8–14.0]) [48]. Glad and colleagues’ [20] also found more pervasive PTSD symptoms predicted subsequent PGD beyond the first year. Long-lasting PTSD symptoms like cognitive and behavioral avoidance [46] may impede emotional processing and prolong separation distress, thereby initiating or prolonging PGD symptoms. Therefore, suffering prolonged PTSD symptoms after a traumatic ICU loss is an important risk factor for PGD.

### Study strengths and limitations

The strength of this study lies in its use of CLPM for four-wave longitudinal data measured between 6 and 24 months post-loss to explore the complex temporal reciprocal relationships between PGD, PTSD, and depressive symptoms for family surrogates who lost a critically ill relative in the ICU. Timely identification and alleviation of depression and PGD symptoms during early bereavement may enable bereaved surrogates to grieve more effectively and avoid long-lasting PGD, PTSD, and depression (via PTSD) (Fig. 2).

However, several study limitations should be recognized. Our study findings may not be generalized to (inter)national populations beyond the sampled hospitals or surrogates who did not participate in or withdrew from bereavement surveys despite no observed differences in baseline characteristics and bereavement data missed completely at random. We may overestimate bereaved surrogates’ psychological distress by measuring PGD, PTSD, and depressive symptoms with screening rather than diagnostic tools, but this measurement strategy may avoid overlooking their need for emotional support. Our measurement of PGD by the PG-11 does not fully assess prolonged grief according to the criteria in DSM-V (PCBD) or ICD-11 (PGD) [49]. Time frame of our assessments of grief reactions limits understanding the temporal reciprocal relationships between PGD, PTSD, and depression beyond the first 2 bereavement years.

## Conclusions

PGD symptoms at 6 months after bereavement may contribute to the exacerbation of PTSD symptoms over time, whereas long-lasting PTSD symptoms disrupt the normal grief process and precipitate prolonged depressive and PGD symptoms beyond the first bereavement year. Healthcare professionals should assess PGD and depression among bereaved surrogates as soon as possible after an ICU death (e.g., 6 months post-loss). Elevated PGD and depressive symptoms early in bereavement can be considered a risk factor for prolonged PTSD. Our novel observation of the bidirectional relationship between symptoms of PGD and PTSD in the second bereavement year indicates that symptoms of PGD and PTSD beyond the first bereavement year may fuel each other, precluding recovery from loss. Targeting elevated PGD and PTSD symptoms may alleviate these grief-related responses.

## Supplementary Information

**Additional file 1**. **Table S1**: Comparisons of family characteristics across participation status during bereavement follow-ups (N = 303).

**Additional file 2**. **Table S2**: Comparisons of psychological distress across participation status during bereavement follow-ups (N =293).

**Additional file 3**. **Table S3**. Comparisons of patient characteristics across participation status during bereavement follow-ups (N = 303).

## Data Availability

The sharing of anonymized data from this study is restricted due to ethical and legal constrictions. Data contain sensitive personal health information, which is protected under The Personal Data Protection Act in Taiwan, thus making all data requests subject to Institutional Review Board (IRB) approval. Per Chang Gung Memorial Hospital (CGMH) IRB, the data that support the findings of this study are restricted for transmission to those in the primary investigative team. Data sharing with investigators outside the team requires IRB approval. All requests for anonymized data will be reviewed by the research team and then submitted to the CGMH IRB for approval.

## Abbreviations

AIC: Akaike information criterion
APACHE: Acute physiology and chronic health evaluation
BIC: Bayesian information criterion
CFI: Comparative fit index
CI: Confidence interval
CLPM: Cross-lagged panel modeling
EOL: End of life
FIML: Full information maximum likelihood
HADS: Hospital Anxiety and Depression Scale
HADS-D: HADS depression subscale
ICU: Intensive care unit
IES-R: Impact of Event Scale—Revised
PTSD: Posttraumatic stress disorder
PGD: Prolonged grief disorder
RMSEA: Root-mean-square error of approximation
SABIC: Sample-size adjusted BIC
SRMR: Standardized root-mean-square residual
TLI: Tucker–Lewis index

## Code availability

Specifications for Mplus codes for statistical analyses are available from the corresponding and the first authors upon reasonable request.
