# Supplementary material for: How symptoms of prolonged grief disorder, posttraumatic stress disorder, and depression relate to each other for grieving ICU families during the first two years of bereavement
Source: Crit Care. 2022 Nov 1;26:336. doi: 10.1186/s13054-022-04216-5 (PMC9628049; doi:10.1186/s13054-022-04216-5)
Supplement: Supplementary file 1 — Additional file 1. Table S1: Comparisons of family characteristics across participation status during bereavement follow-ups (N = 303). [file 13054_2022_4216_MOESM1_ESM.docx]

**Supplemental Table 1.** **Comparisons of family characteristics across participation status during bereavement follow-ups (*N* = 303)^a^**

| Variable | Completed  follow-ups  (*n*=245) | Withdrew from follow-ups  (*n*=39) | Skipped follow-ups (*n*=16) | *P* |
| --- | --- | --- | --- | --- |
| Age, *n* (%) |  |  |  | .954 |
| 21-45 | 99 (40.4%) | 15 (38.5%) | 6 (37.5%) |  |
| 46-55 | 66 (26.9%) | 14 (35.9%) | 5 (31.3%) |  |
| 56-65 | 48 (19.6%) | 6 (15.4%) | 3 (18.8%) |  |
| >65 | 32 (13.1%) | 4 (10.3%) | 2 (12.5%) |  |
| Gender, *n* (%) |  |  |  | .164 |
| Male | 108 (44.1%) | 11 (28.2%) | 6 (37.5%) |  |
| Female | 137 (55.9%) | 28 (71.8%) | 10 (62.5%) |  |
| Marital status, *n* (%) |  |  |  | .541 |
| Single | 53 (21.6%) | 12 (30.8%) | 2 (12.5%) |  |
| Married/Cohabiting | 186 (75.9%) | 26 (66.7%) | 14 (87.5%) |  |
| Separated/Widowed | 6 (2.4%) | 1 (2.6%) | - (0.0%) |  |
| Educational level, *n* (%) |  |  |  | .869 |
| >Senior high school | 122 (49.8%) | 19 (48.7%) | 9 (56.3%) |  |
| ≦Senior high school | 123 (50.2%) | 20 (51.3%) | 7 (43.8%) |  |
| Financial status, *n* (%) |  |  |  | .192 |
| Making ends meet | 208 (84.9%) | 29 (74.4%) | 16(100.0%) |  |
| Financial strain | 32 (13.1%) | 9 (23.1%) | - (0.0%) |  |
| Other | 5 (2.0%) | 1 (2.6%) | - (0.0%) |  |
| Relationship, *n* (%) |  |  |  | .324 |
| Spouse | 74 (30.2%) | 9 (23.1%) | 4 (25.0%) |  |
| Child | 128 (52.2%) | 26 (66.7%) | 11 (68.8%) |  |
| Other | 43 (17.6%) | 4 (10.3%) | 1 (6.3%) |  |
| Chronic disease, *n* (%) |  |  |  | .141 |
| Yes | 89 (36.3%) | 15 (38.5%) | 2 (12.5%) |  |
| No | 156 (63.7%) | 24 (61.5%) | 14 (87.5%) |  |
| Living with the patient, *n* (%) | |  |  | .983 |
| Yes | 163 (66.5%) | 26 (66.7%) | 11 (68.8%) |  |
| No | 82 (33.5%) | 13 (33.3%) | 5 (31.3%) |  |

| Variable | Completed  follow-ups  (*n*=245) | Withdrew from follow-ups  (*n*=39) | Skipped follow-ups (*n*=16) | *P* |
| --- | --- | --- | --- | --- |
| Hospitalization for mental health problems, *n* (%) | | | | $.^{a}$ |
| Yes | - (0.0%) | - (0.0%) | - (0.0%) |  |
| No | 244(100.0%) | 39(100.0%) | 16(100.0%) |  |
| Hospitalization for medical problems, *n* (%) | | | | .939 |
| Yes | 11(4.5%) | 2(5.1%) | 1(6.3%) |  |
| No | 234(95.5%) | 37(94.9%) | 15(93.8%) |  |
| Emergency room visit, *n* (%) | |  |  | .146 |
| Yes | 18 (7.3%) | - (0.0%) | 2 (12.5%) |  |
| No | 227 (92.7%) | 39 (100.0%) | 14 (87.5%) |  |
| Medication use for pain problems, *n* (%) | | |  | .526 |
| Yes | 31 (12.7%) | 3 (7.7%) | 1 (6.3%) |  |
| No | 214 (87.3%) | 36 (92.3%) | 15 (93.8%) |  |
| Medication use for anxiety problems, *n* (%) | | |  | .658 |
| Yes | 6 (2.4%) | 1 (2.6%) | 1 (6.3%) |  |
| No | 239 (97.6%) | 38 (97.4%) | 15 (93.8%) |  |
| Medication use for depressive problems or other psychiatric disturbances, *n* (%) | | | | .712 |
| Yes | 3 (1.2%) | - (0.0%) | - (0.0%) |  |
| No | 242 (98.8%) | 39 (100.0%) | 16 (100.0%) |  |

^a^Among the 303 family surrogates who participated in bereavement surveys, follow-up assessments were not due for 3 participants.

^b^ cannot be estimated.
