# Supplementary material for: How symptoms of prolonged grief disorder, posttraumatic stress disorder, and depression relate to each other for grieving ICU families during the first two years of bereavement
Source: Crit Care. 2022 Nov 1;26:336. doi: 10.1186/s13054-022-04216-5 (PMC9628049; doi:10.1186/s13054-022-04216-5)
Supplement: Supplementary file 2 — Additional file 2. Table S2: Comparisons of psychological distress across participation status during bereavement follow-ups (N =293). [file 13054_2022_4216_MOESM2_ESM.docx]

**Supplemental Table 2. Comparisons of psychological distress across participation status during bereavement follow-ups (*N* =293)^a^**

| Variable |  | Participants |  | Skipped follow-ups |  | Withdrew from follow-ups | *P* |
| --- | --- | --- | --- | --- | --- | --- | --- |
| HADSA, mean (SD) | N |  | N |  | N |  |  |
| Time 6 | 285 | 3.89(3.64) | 8 | 3.63(2.92) |  |  | 0.840 |
| Time 13 | 270 | 2.67(2.80) | 8 | 2.75(1.98) | 14 | 2.07(1.64) | 0.721 |
| Time 18 | 264 | 2.38(2.82) |  |  | 13 | 1.69(1.43) | 0.379 |
| Time 24 | 259 | 2.21(2.30) |  |  | 12 | 3.08(2.87) | 0.205 |
| HADSD, mean (SD) |  |  |  |  |  |  |  |
| Time 6 | 285 | 5.39(4.10) | 8 | 3.50(2.20) |  |  | 0.196 |
| Time 13 | 270 | 4.17(3.65) | 8 | 5.37(3.20) | 14 | 3.57(2.98) | 0.530 |
| Time 18 | 264 | 3.70(3.31) |  |  | 13 | 2.69(1.93) | 0.275 |
| Time 24  IES-R, mean (SD) | 260 | 3.53(2.98) |  |  | 12 | 4.33(3.20) | 0.366 |
| Time 6 | 283 | 8.33(9.47) | 8 | 7.50(8.00) |  |  | 0.806 |
| Time 13 | 270 | 6.05(7.30) | 8 | 5.25(4.06) | 14 | 4.57(3.67) | 0.719 |
| Time 18 | 262 | 4.77(6.57) |  |  | 12 | 1.75(1.54) | 0.113 |
| Time 24 | 260 | 3.72(6.030) |  |  | 12 | 3.08(3.78) | 0.716 |
| PG, mean (SD) |  |  |  |  |  |  |  |
| Time 13 | 266 | 17.08(6.85) | 8 | 21.12(5.30) | 14 | 16.07(3.87) | 0.204 |
| Time 18 | 263 | 15.22(5.79) |  |  | 13 | 13.00(1.95) | 0.168 |
| Time 24 | 260 | 14.44(5.16) |  |  | 12 | 14.08(3.15) | 0.809 |

^a^The number of family surrogates who participated in 3 months postloss survey.

Each psychological-distress measurement was compared to the prior wave of assessment.

The number of surrogates who did not complete the prior wave of assessment was 7, 11, and 1 at 13, 18, and 24 months postloss, respectively.
