# Supplementary material for: How symptoms of prolonged grief disorder, posttraumatic stress disorder, and depression relate to each other for grieving ICU families during the first two years of bereavement
Source: Crit Care. 2022 Nov 1;26:336. doi: 10.1186/s13054-022-04216-5 (PMC9628049; doi:10.1186/s13054-022-04216-5)
Supplement: Supplementary file 3 — Additional file 3. Table S3. Comparisons of patient characteristics across participation status during bereavement follow-ups (N = 303). [file 13054_2022_4216_MOESM3_ESM.docx]

**Supplemental Table 3. Comparisons of patient characteristics across participation status during bereavement follow-ups (*N* = 303)^a^**

| Variable, *n* (%) | Completed follow-ups  (*n* =245) | Withdrew from follow-ups (*n*=39) | Skipped follow-ups (*n* =16) | *P* |
| --- | --- | --- | --- | --- |
| Gender |  |  |  | .905 |
| Male | 155 (63.3%) | 25 (64.1%) | 11 (68.8%) |  |
| Female | 90 (36.7%) | 14 (35.9%) | 5 (31.3%) |  |
| Diagnosis |  |  |  | .785 |
| Cancer | 124 (50.6%) | 17 (43.6%) | 6 (37.5%) |  |
| Chest | 18 (7.3%) | 2 (5.1%) | 1 (6.3%) |  |
| Cardiovascular  Digestive  Kidney  Other | 11 (4.5%)  10 (4.1%)  14 (5.7%)  68 (27.8%) | 2 (5.1%)  1 (2.6%)  2 (5.1%)  15 (38.5%) | 1 (6.3%)  - (0.0%)  - (0.0%)  8 (50.0%) |  |
| Acute symptoms/problems at admission | | |  | .147 |
| Respiratory failure/distress  Infection  Shock  Bleeding  Cardiac arrest  Others | 123 (50.2%)  69 (28.2%)  23 (9.4%)  9 (3.7%)  8 (3.3%)  13 (5.3%) | 25 (64.1%)  7 (17.9%)  1 (2.6%)  1 (2.6%)  3 (7.7%)  2 (5.1%) | 7 (43.8%)  9 (56.2%)  - (0.0%)  - (0.0%)  - (0.0%)  - (0.0%) |  |
| Comorbidity |  |  |  | .931 |
| Yes | 209 (85.3%) | 34 (87.2%) | 14 (87.5%) |  |
| No | 36 (14.7%) | 5 (12.8%) | 2 (12.5%) |  |
| Variable, Mean (SD) |  |  |  |  |
| Age (years) | 66.08 (14.16) | 68.51 (15.17) | 72.06 (14.45) | .191 |
| APACHE^b^ | 28.45 (5.29) | 27.97 (5.35) | 27.00 (6.56) | .531 |
| SOFA^b^ | 12.42 (3.95) | 12.51 (4.00) | 11.50 (5.48) | .665 |
| Length of ICU stay (days) | 20.64 (15.02) | 22.10 (17.89) | 24.75 (14.99) | .528 |
| Time from ICU admission to enrollment (days) | 14.51 (13.02) | 15.23 (10.98) | 17.19 (12.92) | .695 |
| Time from enrollment to death (days) | 7.13 (8.07) | 7.87 (11.31) | 8.56 (7.90) | .734 |

^a^Among the 303 family surrogates who participated in bereavement surveys, follow-up assessments were not due for 3 participants. ^b^Measured at enrollment.
